# Sexuality and Mental Health: The Need for Mutual Development and Research

**DOI:** 10.3390/jcm8111794

**Published:** 2019-10-26

**Authors:** Angel L. Montejo

**Affiliations:** University of Salamanca, Psychiatry Service, Clinical Hospital, Faculty of Nursing and Institute of Biomedical Research of Salamanca (IBSAL), Avenue of Donantes de Sangre SN, 37007 Salamanca, Spain; amontejo@usal.es; Tel.: +34-639754620

**Keywords:** sexuality, mental health, research, sexual dysfunction

## Abstract

Research in the field of sexuality has shown growing scientific development in recent years, although there’s a lack of well-trained professionals who could contribute to increasing its benefits. Sexuality continues to be a taboo with different interpretations and difficult delimitation of either normal or pathological behavior. More resources are needed for the understanding of new emerging pathologies, and to increase the research in new models of sexual behavior. All psychiatric diseases include symptoms affecting sexual life, such as impaired desire, arousal, or sexual satisfaction that need to be properly addressed. Health providers and prescribers must detect and prevent iatrogenic sexual dysfunction that can highly deteriorate a patient’s sexual life and satisfaction, leading to frequent drop-outs of medication. Approaching and researching aspects of sexual intimacy, life desires, frustrations, and fears undoubtedly constitutes the best mental health care.

Sexuality, understood as a drive and an inherent need for human beings, has unquestionably been part of the occupations and concerns of psychiatrists from the beginning of the century. Not in vain, psychoanalysts theorized about the importance of sexual repression as the origin of a great number of mental diseases. Sexual drive, originally called libido, seemed to be the nucleus of life and its repression or deficiencies a way towards mental suffering. The concept obviously must be extended towards eroticism in a broader sense, not always necessarily coital, and to satisfaction of physical pleasure and intimacy. Over the years, following growth in scientific research, it has become essential to invest increasing interest and more research resources to contribute to the theoretical maxims that could empirically explain the secrets of such important drives.

Fortunately, research in the field of sexuality has shown growing scientific development, leading to the greater interest of researchers [1,2,3,4,5,6,7,8]. The emergence of an increasing number of specific journals focusing on some either large or small sexual issues are symptomatic of our contemporary society’s concerns. The great and unexpected role of sexual abuse in the origin or development of some mental illnesses and the boundaries between normal and pathological sexuality, without having so far found satisfactory agreement in this sense, have constituted some of the areas of greatest interest.

However, one of the biggest limitations for the generalization of adequate sexual health is the lack of well-trained professionals who could contribute to increasing its benefits. The training of mental health providers focusing in sexology has not developed accordingly to accompany the population’s needs. Sexuality continues to be a taboo, and professionals dealing with its research and treatment remain scarce, even with a large heterogeneous background. The widespread access to continuous, multiple, and often unhealthy sexual content without any ethical filter or prior preparation in our young people has been a new challenge in addressing their understanding. The different interpretations of such a variable concept leads to an extremely difficult delimitation of either normal or pathological sexuality. The easy to use and generalized online access has popularized sexual performance so much that some new unexpected phenomena have recently emerged, such as online shared group rapes or the increased number of “unlinked sexual seekers” looking for some new variate, intense, and prolonged sexual experiences, as well as some novel shocking sensations such as chemsex. More resources are needed to cope with the appearance of these new emerging pathologies, and to increase the research in these new models of sexual behavior. Unfortunately, in most parts of the world, basic training in sexology has not been sufficiently developed as a fundamental part of the scientific growth of our mental health professionals. Sexuality is commonly interpreted as a minor discipline that unfortunately is not included as a part of the basic training to provide adequate support for normal subjects and mental health patients.

It is well known that all psychiatric diseases include some variations in sexual symptoms and difficulties with highly different individual sexual meanings and concerns. Depression, bipolar disorder, anxiety disorders, or even psychosis include symptoms affecting sexual life, such as impaired desire, arousal, or sexual satisfaction that inevitably need to be properly identified and addressed. There are no sexless human beings, and neither are our patients sexless, even if they do not carry out an active sex life.

As a main classification instrument today, the Diagnostic and Statistical Manual of Mental Disorders DSM-5 recognizes certain sexual conditions to which it grants diagnostic criteria, although not without some controversy. It would be very unfortunate if this would be the only approach to bringing the average professional closer to the sexual life and intimacy needs of their patients. These days, hypoactive sexual desire or even aversion to sex (paradoxically frequently iatrogenic after the prescription of a chronic use of serotonergic antidepressants) have reached almost epidemic proportions that remain unnoticed and understudied. Additionally, there is a lack of economic resource investment in their research by the pharmaceutical companies themselves or by public health systems. Generally, a great number of antidepressant prescribers are poorly motivated to detect and prevent iatrogenic sexual dysfunction that can highly deteriorate the patient’s sexual life and satisfaction, leading to subsequent emotional deprivation of all those who must endure it in the medium and long term, as serotonergic antidepressants (SSRIs) remain the most prescribed in the Western world. 

Taking into account the patients with psychosis, there may be some clinicians who consider that it would be better not to investigate the sexual life of their patients, as this could worsen psychotic symptoms, or simply interpret that the information obtained would be unreliable. Many others may avoid it, because in this way they are not forced to face the side effects of some prescribed antipsychotics that intensely block the dopamine activity and deteriorate sexual functioning. Let us remember that sexuality includes the creation of links and intimacy with another person, which helps patients to fight against the negative symptoms of the disease. Perhaps some clinicians consider that sexual relationships in psychiatric female patients with chronic psychosis mainly involves a risk of pregnancy and the appearance of sexually transmitted diseases. Therefore, implicitly, the absence of any interview about their sexual life and interpersonal relationships, including the needs of intimacy and maternity plans, promotes a silent sterilization. That is, the prescription of an antipsychotic that increases prolactin blood levels is inevitably linked to anovulation and sterility. Can patients then decide on their motherhood? Obviously not, because often those who prescribe these antipsychotics have inappropriately decided for their patients without exchanging a single comment or adequate reflection about their family life project. On the other hand, some HIV-positive patients are severely mentally ill and use prostitution as the only means of obtaining sexual pleasure and intimacy. Most of these patients have limited stable sexual relationships or sex partners, and many of them have none except masturbation, duplicating the general population rates of prostitution and the consequent increased risks of HIV and sexually transmitted diseases. Perhaps some may think that these are issues outside the mental health professional’s goal and that they would be much better addressed by other health providers; however, unfortunately, these patients go to a general practitioner infrequently, and rarely establish lasting and close relationships with them. In addition, frequent drop-outs of medication have been reported due to iatrogenic sexual dysfunction associated with the use of hyperprolactinemic antipsychotics, which remains widely underestimated by psychiatrists despite its striking clinical implications in the short, medium, and long term. The abrupt or progressive decline in desire, excitatory, and/or orgasmic function compromises the compliance and makes long-term treatment uncertain in some specific groups, such as young male patients. The emphatic approach to this adverse event by clinicians, through adequate sensitization and training, would prevent catastrophic consequences compromising the clinical evolution of patients with psychosis and, moreover, improve the doctor–patient relationship. Approaching the aspects of intimacy, life desires, frustrations, and fears undoubtedly constitutes real mental health care. As a sample of this, in a recent survey on sexual health in Spain [9] a large number of people were interviewed about their motivation for sexual intercourse. Surprisingly, only a few of them selected sexual pleasure as a fundamental reason (mostly males) or procreation (mostly women). The vast majority pointed out that the main reason was the search for emotional intimacy or to satisfy the need to love and be loved. However, sexual pleasure is once again only a small part of love.

For many of us, it is never too late to regain the study and approach to sexuality and its concerns as something enriching in mental health, and of course in the global existence of our patients. We may not need to be sexologists but recovering sexuality as a basic aspect of mental health must become one of our most current aims. The way forward must be through the incorporation of sexuality as an inseparable part of the human being and its research as an essential instrument in the holistic vision of the existence of our patients.
